# 

**Published:** 1897-06

**Authors:** 


CONTENTS—JUNE, 1897.—VOL. XII., NO. 12.

ORIGINAL CONTRIBUTIONS—

Case of Mostoiditis Complicating Purulent Otitis Media Cured by Enlarging the Drum; Perforation and Syringing the Tympanitic Cavity. W. S. Scheppegrell, A. M., M. D. . . . . . . . . . . . . . . . . . . . . . 653
Water the Source of Malarial Poison. J. E. Gardiner, M. D. . . . . . . . . 655
Medical Education in Texas. James Orr, M. D. . . . . . . . . . . . 664
A Case of Foreign Bodies in the Stomach, with Treatment. James S. Watson, M. D. . . . . . . . . . . . . . . . . . . . . . . . . . 666
Public Hygiene—Resolutions and Suggestions. W. Neal Watt, M. D. . . 667
Biographical—Prof. Bacon Saunders, M. D., President Texas State Medical Associations . . . . . . . . . . . . . . . . . . . . . . . . . 669

SOCIETY NOTES—

Northwest Texas Medical Association . . . . . . . . . . . . . . . . . . . 670
New York Academy of Medicine—Section on Orthopædic Surgery . . . . 676
Brazos Valley Medical Association . . . . . . . . . . . . . . . . . . . 678
South Texas Medical Association . . . . . . . . . . . . . . . . . . . . 682

ABSTRACTS AND SELECTIONS—

Asexualization for Crime . . . . . . . . . . . . . . . . . . . . . . . 684

EDITORIAL—

Is Malaria a Land or Water Disease . . . . . . . . . . . . . . . . . . 687
Cheap Learning . . . . . . . . . . . . . . . . . . . . . . . . . . . . 688
Veto of the Divorce Bill . . . . . . . . . . . . . . . . . . . . . . . 689

MEDICAL NEWS AND MISCELLANY . . . . . . . . . . . . . . . . . . 691-694
BOOK NOTICES . . . . . . . . . . . . . . . . . . . . . . . . . . . 694-696
PUBLISHERS' NOTES . . . . . . . . . . . . . . . . . . . . . . . . . 696-

